# Evaluation of the Presence and Levels of Enrofloxacin, Ciprofloxacin, Sulfaquinoxaline and Oxytetracycline in Broiler Chickens after Drug Administration

**DOI:** 10.1371/journal.pone.0166402

**Published:** 2016-11-15

**Authors:** Débora Cristina Sampaio de Assis, Guilherme Resende da Silva, Isabela Pereira Lanza, Ana Cláudia dos Santos Rossi Ribeiro, Ângela Maria Quintão Lana, Leonardo José Camargos Lara, Tadeu Chaves de Figueiredo, Silvana de Vasconcelos Cançado

**Affiliations:** 1 Departamento de Tecnologia e Inspeção de Produtos de Origem Animal, Escola de Veterinária, Universidade Federal de Minas Gerais (UFMG), Belo Horizonte, Minas Gerais, Brazil; 2 Laboratório Nacional Agropecuário (LANAGRO-MG), Ministério da Agricultura, Pecuária e Abastecimento, Pedro Leopoldo, Minas Gerais, Brazil; Mayo Clinic Arizona, UNITED STATES

## Abstract

The depletion times of enrofloxacin and its metabolite ciprofloxacin as well as sulfaquinoxaline and oxytetracycline were evaluated in broiler chickens that had been subjected to pharmacological treatment. The presence and residue levels of these drugs in muscle tissue were evaluated using an ultra-performance liquid chromatography-tandem mass spectrometry (UPLC-MS/MS) method that was validated in this work. The results showed the presence of all antimicrobial residues; however, the presence of residues at concentrations higher than the drugs’ maximum residue limit (MRL) of 100 μg kg^-1^ was found only during the treatment period for oxytetracycline and until two days after discontinuation of the medication for enrofloxacin, ciprofloxacin and sulfaquinoxaline. It was concluded that the residues of all antimicrobials were rapidly metabolized from the broiler muscles; after four days of withdrawal, the levels were lower than the limit of quantification (LOQ) of the method for the studied analytes.

## Introduction

The antimicrobials enrofloxacin, sulfaquinoxaline and oxytetracycline have activity against a broad spectrum of Gram-negative and Gram-positive bacteria. These antimicrobials are widely used in poultry production as therapeutic or prophylactic agents because broiler chickens are raised in intensive industrial farming systems, the stressful conditions of which can make these animals more susceptible to infectious diseases [1,2]. However, the indiscriminate use of antimicrobials may lead to the presence of their residues in food products of animal origin. These residues may have adverse health effects in humans, such as allergic reactions, and may lead to selection for bacterial strains that are resistant to antibiotics, especially when there is a lack of compliance with the withdrawal period determined for each medication [2–8].

The study and determination of the appropriate withdrawal period for antibiotics used in the treatment of broiler chickens have economic impacts and influence breeding management, especially in the final stages of broiler production. However, most published studies that have evaluated the pharmacokinetics or the withdrawal period of enrofloxacin in broiler chickens used microbiological assays [9–11] or high-performance liquid chromatography (HPLC) methods [12–14]. These studies also did not employ the quantitative and confirmatory methods recommended by regulatory agencies for the study of veterinary drugs in animal tissues, such as liquid chromatography-tandem mass spectrometry (LC-MS/MS). Moreover, certain studies used only small numbers of experimental animals and did not simulate the breeding conditions used in poultry production [13,15]; such conditions might impose stress that can influence the pharmacokinetic characteristics of the administered drugs. Regarding oxytetracycline, there is a lack of papers in the scientific literature on the evaluation of its depletion kinetics in broiler chickens; the papers that are available in the scientific literature evaluated the depletion time of this antibiotic only in other species [16–20]. Similarly, no published reports employed LC-MS/MS to evaluate the depletion time of sulfaquinoxaline used as a single drug; the information found refers only to this drug’s use in combination with trimethoprim [21].

The establishment of withdrawal periods that are based on scientific studies of the depletion times of veterinary drugs allows appropriate animal treatment and the slaughter of treated animals in compliance with the required period for the elimination of each drug and its residues from the animals’ body.

To ensure human food safety, in addition to establishing compliance with withdrawal periods, maximum residue limits (MRLs) for antimicrobial residues in several animal tissues have been set based on the acceptable daily intake (ADI) for each drug [22]. Regulation No. 37/2010 of the European Commission established MRLs for several pharmacologically active substances in foods of animal origin [23]. To ensure compliance with this regulation, sensitive and specific analytical methods are necessary [24].

Analytical methods based on ultra-performance liquid chromatography (UPLC) techniques coupled to mass spectrometry are indicated for confirmatory studies of antimicrobials because of these methods’ high selectivity and particularly high sensitivity [24,25–27].

Thus, the purpose of the present study was evaluate the depletion times of enrofloxacin and its metabolite ciprofloxacin as well as sulfaquinoxaline and oxytetracycline by studying their presence and residue levels in the muscle tissue of broiler chickens that had been treated with the drugs via drinking water. This evaluation was performed by using UPLC-tandem mass spectrometry (UPLC-MS/MS), which was preliminarily validated in this study to ensure the reliability of the results.

## Materials and Methods

### Chemicals and reagents

The analytical standards enrofloxacin, ciprofloxacin, sulfaquinoxaline and oxytetracycline that were used in the validation were purchased from Sigma-Aldrich and Fluka (St. Louis, MO, USA).

Acetonitrile (LC-MS grade) from Merck (Darmstadt, Germany), trichloroacetic acid (TCA) from J.T. Baker (Center Valley, PA, USA) and heptafluorobutyric acid (HFBA) from Fluka (St. Louis, MO, USA) were used.

### Standard solutions

The stock standard solutions of enrofloxacin (125 μg mL^-1^), ciprofloxacin (125 μg mL^-1^), sulfaquinoxaline (250 μg mL^-1^) and oxytetracycline (200 μg mL^-1^) were prepared by dissolving each standard in methanol. The solutions were then stored at -20°C for up to five months. The working mixed standard solution was diluted with ultrapure water (0.5 μg mL^-1^) and remained stable for one week when stored at -20°C.

### Extraction procedure

Two-gram samples of muscle (thigh and breast) were weighed in 50 mL polypropylene centrifuge tubes, and the analytes were extracted with 8 mL of 5% TCA solution. Each resulting mixture was immersed in an ultrasonic bath for 10 min and then mixed in an orbital shaker for 10 minutes. Then, aliquots of 1.5 mL were transferred to centrifuge microtubes and centrifuged (14.462 x g) at 4°C for 10 min in a refrigerated centrifuge (SIGMA 3-30KS^®^, ATR, Laurel, MD, USA). After centrifugation, the supernatant from each microtube was filtered through a filter unit with a polytetrafluoroethylene (PTFE) membrane (pore size of 0.2 μm, diameter of 13 mm, Millex^®^, Merck Millipore, Darmstadt, Germany), and the filtrate was transferred to a vial for injection.

### Instrumentation

The experiments were performed on a UPLC system (Prominence Shimadzu, Milton Keynes, United Kingdom) coupled to a 4000 QTRAP^®^ mass detector (AB Sciex, Darmstadt, Germany) set in positive ESI mode. An Agilent Zorbax Eclipse XDB C18 (4.6 x 30 mm x 3.5 μm) with a vanguard column was used for chromatographic separation. Mobile phase A consisted of water with 0.2% HFBA, and acetonitrile was used as mobile phase B. The flow rate was set to 0.6 mL min^-1^, and the column temperature was set to 35°C. The initial conditions were set to 10% B, with a linear gradient from 10% B to 50% B from 0.01 to 3 min, respectively. Then, 50% B was maintained for 0.5 min, followed by an immediate return to 10% B at 4 min, which was held for 2 min. The total run time was thus 6 min, and the partial loop with needle overfill injection volume was 10 μL.

### Validation of the analytical method

The UPLC-MS/MS method for the evaluation of enrofloxacin, ciprofloxacin, sulfaquinoxaline and oxytetracycline in broiler chicken muscle was validated by assessing the following performance parameters: linearity, selectivity, precision, accuracy, limit of detection (LOD), limit of quantification (LOQ), decision limits (CCα) and detection capability (CCβ) [24,28,29].

To evaluate linearity, blank samples were spiked with the standard solution of the antibiotics at a concentration of 5 μg kg^-1^, 10 μg kg^-1^, 50 μg kg^-1^, 75 μg kg^-1^, 100 μg kg^-1^, 125 μg kg^-1^ or 150 μg kg^-1^, corresponding to respective values of 0.05, 0.1, 0.5, 0.75, 1.0, 1.25 and 1.5 times the MRL established by European Commission Regulation (EU) No. 37/2010 [10] for the studied analytes, in five replicates. Then, a plot relating peak area to concentration was created to determine the curve equations. The coefficients of determination (R^2^) and correlation (r) were determined by linear regression [23,24,29].

Selectivity was assessed by verifying the presence or absence of interfering compounds eluting at the same retention times as for the analytes of interest [24].

The precision of the method was assessed based on the relative standard deviation (RSD), determined under repeatability and within-laboratory reproducibility conditions. The repeatability of the method was assessed by analysis of blank samples spiked with all of the analytes at each of the three specified levels (50 μg kg^-1^, 100 μg kg^-1^ and 150 μg kg^-1^, corresponding to 0.5, 1.0 and 1.5 times the MRL, respectively) in three replicates (n = 9). The within-laboratory reproducibility was determined by following the same protocol, but with two different operators performing the analysis (n = 18) [24, 28].

The evaluation of the accuracy was performed using recovery tests. For this purpose, blank samples were spiked with the standard solution of the studied antibiotics 10 min before the extraction procedure to simulate natural contamination. Analyses were performed in triplicate, considering the linear interval at three levels: low (0.5 times the MRL, or 50 μg kg^-1^), average (1.0 times the MRL, or 100 μg kg^-1^) and high (1.5 times the MRL, or 150 μg kg^-1^). The recovery obtained at each concentration was calculated using the equation R = (C_1_ / C_2_) x 100, which considers the measured content (C_1_) and the fortification level (C_2_) [24, 30].

CCα and CCβ were calculated by analysis of 20 blank samples spiked at a concentration of 100 μg kg^-1^ (the MRL). The CCα (α = 5%) and CCβ (β = 5%) values were obtained using the equations CCα = MRL + 1.64 x s and CCβ = CCα + 1.64 x s, respectively, which consider the concentration at the permitted limit (MRL) for each analyte and the standard deviation (s) of the within-laboratory reproducibility obtained at the MRL (100 μg kg^-1^) [24].

The LOD and LOQ were calculated using equations that consider the parameters of the analytical curve, namely, LOD = [(3.3 x s)/S] and LOQ = [(10 x s)/S], respectively, using the linear coefficient (s) and the slope (S) of the analytical curve [28].

### Experimental animals

For the depletion studies, 240 1-day-old Cobb chicks, obtained from a commercial hatchery (Pif Paf Alimentos, Minas Gerais, Brazil), were used. The chickens were housed in pens that contained 30 birds each (10 birds/m^2^) and were provided *ad libitum* access to water and non-medicated feed. The chickens were randomly allocated into four experimental groups, labeled from A to D, containing 80 birds each. Chickens in group A formed the untreated control group, whereas those in groups B, C and D were treated with 10 mg kg^-1^ bw of enrofloxacin, sulfaquinoxaline or oxytetracycline, respectively, which was administered via drinking water from the 32^nd^ to 34^th^ day of breeding.

Before the initiation of treatment at the 32^nd^ day, six birds from each group were slaughtered to collect thigh and breast muscle. Then, at days 33, 34, 35, 36, 38, 40, 42, 44 and 46 of breeding, an additional six birds from each group were slaughtered, and samples were again collected. The samples were individually collected and stored at -20°C for UPLC-MS/MS analysis using the previously validated method.

This study was carried out in strict accordance with the recommendations of the National Council for the Control of Animal Experimentation (CONCEA) at the Brazilian Ministry of Science and Technology and Innovation (MCTI). The protocol was approved by the Ethics Committee in Animal Experimentation at the *Universidade Federal de Minas Gerais* (UFMG) (Permit Number: 93/2015). The broiler chickens were euthanized according to the principles of humane slaughter and were properly stunned using the method of electrical stunning before bleeding to minimize suffering.

### Statistical analysis

To assess the depletion times of the antimicrobials in broiler chickens, the experiment was conducted using a completely randomized, 4x10 factorial design (4 antimicrobials x 10 days of evaluation) with 6 replicates. The antimicrobial levels were subjected to nonparametric analyses using the Kruskal-Wallis test at a 5% significance level.

## Results and Discussion

### Mass spectrometry optimization

The operational conditions of the mass spectrometer were established by direct infusion of the standards in the presence of the mobile phase. The capillary voltage was set at 5.5 kV, and the temperature of the source was set at 650°C. Nitrogen was used as the collision gas at 8.0 psi and as the curtain gas at 20.0 psi. The declustering potential (DP) and the collision energy (CE) were optimized for each analyte to improve the signal intensity. Two multiple reaction monitoring (MRM) transitions were established and monitored for each analyte. The relative ion intensity of each studied analyte was evaluated and found to be adequate according to the criteria established by European Commission Decision 2002/675/EC [24] (Table 1).

**Table 1:** MRM transitions and MS/MS parameters used in the validation for each analyte.

Analyte	DP^a^ (V)	Precursor ions (m/z)	CE^b^ (eV)	Product ions (m/z)	CXP^c^ (V)	RT^d^ (min)	Relative intensity
Enrofloxacin	72	360.0	30	342.0	12	3.26	19.78±1.6
	72	360.0	50	286.0	12	3.26	19.78±1.6
Ciprofloxacin	61	332.0	30	314.0	12	3.08	55.55±4.3
	61	332.0	47	231.0	12	3.08	55.55±4.3
Sulfaquinoxaline	50	301.0	23	156.0	12	3.56	34.75±1.8
	50	301.0	40	108.0	12	3.56	34.75±1.8
Oxytetracycline	41	461.3	59	201.1	12	3.13	75.29±9.1
	41	461.0	53	283.2	12	3.13	75.29±9.1

^a^DP: declustering potential

^b^CE: collision energy

^c^CXP: c*ollision* cell exit potential

^d^RT: retention time

### Validation study

Prior to the evaluation of the residues in the broiler chicken muscle, validation of the UPLC-MS/MS analytical method was performed. This is a requirement of the *Codex Alimentarius* for analytical methods used in residue control programs, ensuring the reliability of reported values [30]. Evaluation of the performance parameters demonstrated the complete adequacy of the method for detecting and quantifying the residues of enrofloxacin, ciprofloxacin, sulfaquinoxaline and oxytetracycline in the broiler chicken muscle.

**Fig 1:**
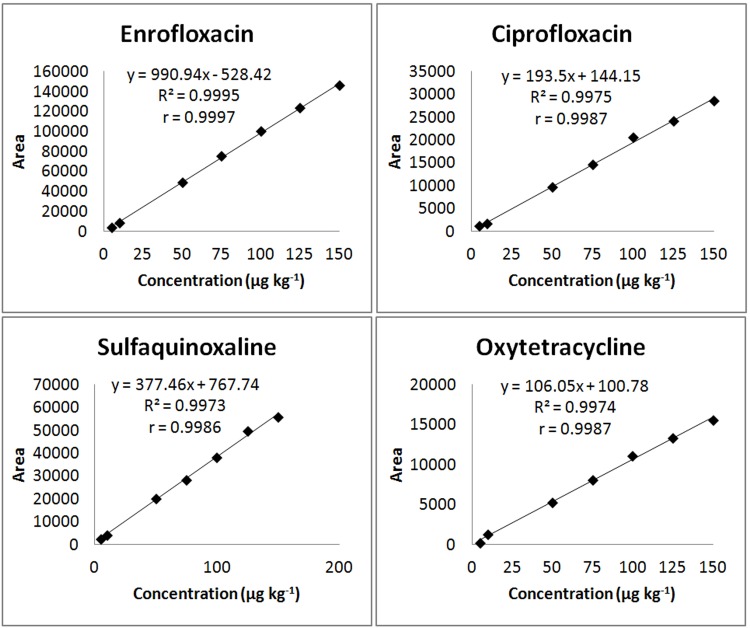
Linearity curves of the antibiotics enrofloxacin, ciprofloxacin, sulfaquinoxaline and oxytetracycline when added to the broiler chicken muscle matrix at a concentration of 5.0, 10.0, 50.0, 75.0, 100.0, 125.0 or 150.0 μg kg^-1^.

The equations of the curves and the coefficients of determination (R^2^) and correlation (r), which were used to evaluate the linearity in the range from 5.0 to 150 μg kg^-1^, are presented in Fig 1. The model is adequate, as the R^2^ values of the analytical curves were higher than 0.99, indicating a good fit of the data to the regression line. Values higher than 0.99 for linearity tests are recommended by the European Commission and INMETRO [24,29]. The estimation of R^2^ provides an evaluation of the quality of the curve obtained, given that the closer its value is to 1.0, the lower the dispersion and uncertainty of the set of experimental points are [31].

**Fig 2:**
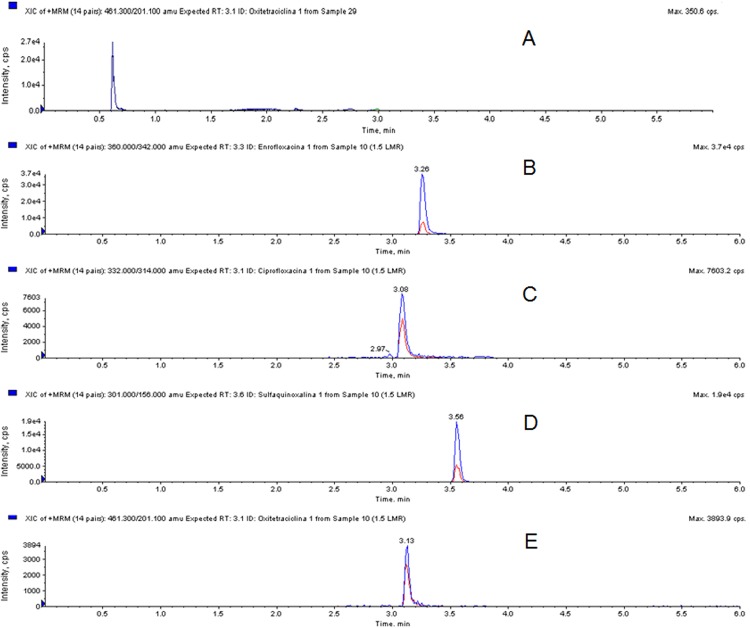
Chromatogram of a muscle sample without the addition of antibiotic standard solution (A) and MRM transitions of the antibiotics enrofloxacin (B), ciprofloxacin (C), sulfaquinoxaline (D) and oxytetracycline (E) when monitored in the broiler chicken muscle matrix.

To evaluate the selectivity of the method, the presence or absence of interfering compounds eluting at the same retention times as for the analytes of interest was verified. The analyses of blank muscle samples (without the addition of antibiotic standard solution) demonstrated that there were no interfering compounds from the extraction procedure that eluted at the same retention times as for the analytes of interest, demonstrating the selectivity of the method. The retention times were 3.26 min for enrofloxacin, 3.08 min for ciprofloxacin, 3.56 for sulfaquinoxaline and 3.13 min for oxytetracycline (Fig 2).

The precision of the method was assessed under repeatability conditions (analyst 1) and within-laboratory reproducibility conditions (analyst 1 + analyst 2) and exhibited adequate results in the working range for all of the analytes studied. The RSD values of the results obtained under repeatability conditions ranged from 1.46 to 18.44%. These values are in accordance with those established by the European Commission [24], which recommends a maximum RSD of 20% for analyte concentrations ranging from 5 to 100 μg kg^-1^ and a maximum RSD of 15% for analyte concentrations from 100 to 1000 μg kg^-1^. Under within-laboratory reproducibility conditions, the RSD values ranged from 3.97 to 16.27% in the present study. According to the *Codex Alimentarius* [30], the maximum RSD values obtained under within-laboratory reproducibility conditions are 32%, 22% and 18% for analyte concentrations from 1 to 10, 10 to 100 and 100 to 1000 μg kg^-1^, respectively (Table 2).

**Table 2:** Values of recovery, relative standard deviation (RSD), limits of detection (LOD) and quantification (LOQ), decision limits (CCα) and detection capability (CCβ) obtained in the validation experiments.

Antibiotic	Level (μg kg^-1^)	REC%^a^	RSD% Repeat^b^	RSD% Repro^c^	LOD^d^ (μg kg^-1^)	LOQ^e^ (μg kg^-1^)	CCα^f^ (μg kg^-1^)	CCβ^g^ (μg kg^-1^)
**Enrofloxacin**								
	50	97.31	18.44	13.52	1.76	5.33	111.70	123.40
	100	98.89	14.16	11.39	1.76	5.33	111.70	123.40
	150	95.89	13.52	12.13	1.76	5.33	111.70	123.40
**Ciprofloxacin**								
	50	104.90	8.10	9.41	2.46	7.45	120.86	141.72
	100	108.30	1.46	8.18	2.46	7.45	120.86	141.72
	150	108.03	4.43	3.98	2.46	7.45	120.86	141.72
**Sulfaquinoxaline**								
	50	100.23	3.30	16.27	6.71	20.34	111.34	122.67
	100	106.28	7.76	8.16	6.71	20.34	111.34	122.67
	150	102.64	5.85	3.97	6.71	20.34	111.34	122.67
**Oxytetracycline**								
	50	88.87	17.79	14.30	3.12	9.46	113.22	126.43
	100	94.26	6.05	8.01	3.12	9.46	113.22	126.43
	150	91.93	5.37	4.80	3.12	9.46	113.22	126.43

^a^REC% = recovery

^b^RSD% Repeat = relative repeatability standard deviation

^c^RSD% Repro = relative within-laboratory reproducibility standard deviation

^d^LOD = limit of detection

^e^LOQ = limit of quantification

^f^CCα = decision limits

^g^CCβ = detection capability

The accuracy was adequate for all of the studied analytes, with mean recovery values of 97.36%, 107.08%, 103.05% and 86.40% for enrofloxacin, ciprofloxacin, sulfaquinoxaline and oxytetracycline, respectively (Table 2). These values are consistent with the reference parameters established by the *Codex Alimentarius* [30].

The CCα and CCβ values were calculated based on analyses of 20 blank samples fortified at the MRL and are presented in Table 2. CCα indicates the limit at and above which it can be concluded, with an error probability of α (5%), that a sample is non-compliant, whereas the CCβ value indicates the smallest content of the substance that can be detected, identified and/or quantified in a sample with an error probability of β (5%) [24].

The LOD of the method varied from 1.76 to 6.71 μg kg^-1^ for the studied analytes, whereas the LOQ varied from 5.33 to 20.34 μg kg^-1^ (Table 2). The obtained values were significantly lower than the MRL established for each analyte; therefore, this method may be used to accurately and reliably monitor residue concentrations lower than those allowed by Regulation (EU) No. 37/2010 [23].

The extraction procedure in proposed method consists only of protein precipitation with TCA. Because of this, compared to other methods described in the literature, which are expensive and require more analysis time [32], the analytical method validated in this work presents the advantage of simpler and faster sample preparation and allows identification and quantification of the studied analytes in a single analytical run with a total run time of 6 min.

### Depletion study

Veterinary drugs are administered to food-producing animals for therapeutic purposes. It is important to assess the depletion times of veterinary drugs in edible tissues to ensure public health because the presence of such substances in foods of animal origin at concentrations higher than the MRL established for each drug may lead to toxicity, allergic reactions and/or selection for bacterial strains that are resistant to antibiotics.

Residues of enrofloxacin and its metabolite ciprofloxacin as well as sulfaquinoxaline and oxytetracycline were not detected in any of the samples of broiler chickens from the control group (group A), indicating that there was no contamination of the feed and no cross-contamination during the treatments.

In the group of chickens treated with enrofloxacin (group B), the highest concentrations of this drug and its metabolite ciprofloxacin were found at 33, 34, 35 and 36 days of age (P<0.05) (Table 3).

**Table 3:** Mean concentrations of residues of enrofloxacin and ciprofloxacin, as analyzed by UPLC-MS/MS in the broiler chicken muscle matrix, according to the day of breeding.

Day of breeding	Enrofloxacin (μg kg^-1^)	Ciprofloxacin (μg kg^-1^)	Sulfaquinoxaline (μg kg^-1^)	Oxytetracycline (μg kg^-1^)
32	ND^a^ a	ND^a^ a	ND^a^ a	ND^a^ a
33	2941.763 b	241.702 b	4565.472 b	105.913 b
34	2994.004 b	273.462 b	5428.415 b	106.106 b
35	2551.823 b	358.840 b	3438.690 a	61.156 a
36	484.127 b	44.570 b	539.364 a	45.044 a
38	<LQ^b^ a	<LQ^b^ a	<LQ^b^ a	<LQ^b^ a
40	6.227 a	ND^a^ a	ND^a^ a	ND^a^ a
42	<LQ^b^ a	ND^a^ a	ND^a^ a	ND^a^ a
44	6.789 a	ND^a^ a	ND^a^ a	ND^a^ a
46	<LQ^b^ a	ND^a^ a	ND^a^ a	ND^a^ a

Means followed by different letters differed significantly according to the Kruskal-Wallis test (P<0.05).

^*a*^ND: <1.76 μg kg^-1^ (enrofloxacin), <2.46 μg kg^-1^ (ciprofloxacin), <6.71 μg kg^-1^ (sulfaquinoxaline) and <3.12 μg kg^-1^.

^*b*^LOQ: limit of quantification = 5.33 μg kg^-1^ (enrofloxacin), 7.45 μg kg^-1^ (ciprofloxacin), 20.34 μg kg^-1^ (sulfaquinoxaline) and 9.46 μg kg^-1^ (oxytetracycline).

**Fig 3:**
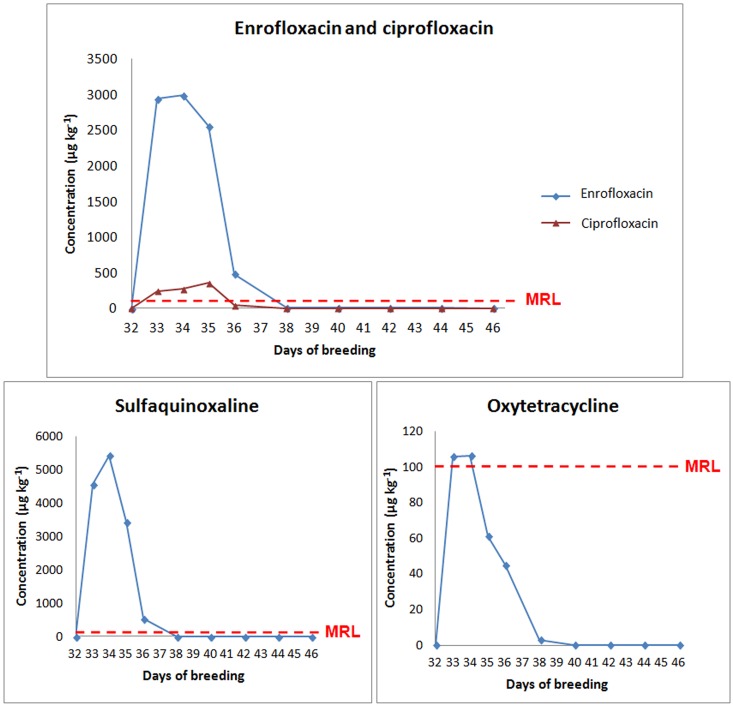
Antimicrobial residues in muscle tissues from broiler chickens subjected to a pharmacological treatment with enrofloxacin, sulfaquinoxaline and oxytetracycline during the drug administration period and in the following days of residue evaluation.

After the initiation of drug administration via drinking water, a rapid increase in the levels of enrofloxacin residues was observed. The treatment was discontinued at the 34^th^ day, and the concentration of enrofloxacin declined rapidly. Ciprofloxacin showed similar characteristics; however, at the 35^th^ day, although enrofloxacin content was reduced, an increase in the concentration of ciprofloxacin was observed because ciprofloxacin is a metabolite of enrofloxacin (Fig 3).

Residue concentrations of enrofloxacin that were higher than the MRL (100 μg kg^-1^) were found in the muscle samples until the 36^th^ day of breeding, i.e., until two days after the end of treatment. Despite insufficient data on residue depletion in the scientific literature, the European Medicines Agency (EMA) recommends a 3-day meat withdrawal period for enrofloxacin used in the treatment of chickens at a dose of 10 mg kg^-1^ bw [33]. After fluoroquinolones are administered, they are rapidly absorbed in the gastrointestinal tract. Enrofloxacin has high bioavailability and a high volume of distribution [34]. However, the metabolism of enrofloxacin in broiler chickens and the accumulation of this drugs in the tissues are relatively low, which may explain the short withdrawal period prior to the slaughter of broiler chickens.

According to published data, administered enrofloxacin is excreted in the form of both the parent compound and the main metabolite, ciprofloxacin; however, the metabolism of enrofloxacin to ciprofloxacin in poultry has been reported to be lower than in pigs or cattle [35].

The clearance mechanisms and elimination pathways for fluoroquinolones have not been explicitly determined in broiler chickens, but residues of parent fluoroquinolones and metabolites are found in both the liver and the kidney after oral administration to chickens [36–38]. While the exact clearance mechanisms for enrofloxacin in broiler chickens have not been determined in the literature, it is likely that enrofloxacin is cleared by both renal and hepatic pathways, as the same applies for its metabolite ciprofloxacin [34,39].

Although enrofloxacin is only used in veterinary medicine, its metabolite ciprofloxacin is a pharmacologically active antimicrobial used in human medicine [15]. The United States prohibits the use of enrofloxacin in food-producing animals [40], and its application is not indicated for laying hens in the EU [23]. The Food and Drug Administration (FDA) suspended approval for the use of enrofloxacin in poultry in 2005, as the use of fluoroquinolones in poultry can lead to the development of fluoroquinolone-resistant *Campylobacter* spp. and consequently increase the infection rate in humans [41]. Because of this, the FDA imposed a zero-tolerance policy for residues of enrofloxacin and ciprofloxacin in broilers, with the target tissue used for monitoring these residues being muscle; this is the tissue with the greatest antibiotic concentration and persistence, with the highest concentration of residues found in the breast muscle [11].

Similar to the results observed for enrofloxacin, concentrations of sulfaquinoxaline that were higher than the drug’s MRL (100 μg kg^-1^) were found until the 36^th^ day of breeding in the current study (Table 3). At the 38^th^ day of breeding, the residue levels were lower than the LOQ of the method (Fig 3).

There is a lack of information in the literature about the depletion time of sulfaquinoxaline in broiler chickens. Lim et al. (2015) evaluated the use of sulfaquinoxaline in combination with trimethoprim in broilers, and the withdrawal time was suggested to be over 5 days after cessation of the medication [21]. Parameters such as drug dose and the route of administration may interfere with the pharmacokinetic properties of drugs and consequently with their depletion time.

After oral administration, sulfonamides are rapidly absorbed in the gastrointestinal tract; 70% to 100% of the oral dose is absorbed, and the drugs are distributed to all tissues [42]. Drug elimination is mainly via feces and urine; therefore, the habit of coprophagy among broilers may increase the depletion time of the drugs, supporting a withdrawal period of 10 days [43]. However, in the present study, the need for compliance with this long withdrawal period was not evident because concentrations lower than the LOQ of the method were observed by the fourth day after the end of treatment.

In contrast with the results observed for enrofloxacin and sulfaquinoxaline, residue levels of oxytetracycline at concentrations higher than the MRL (100 μg kg^-1^) were only observed during the treatment period, until the 34^th^ day of breeding (Table 3 and Fig 3). Oxytetracycline is the least lipophilic member of the tetracycline group and consequently has a lower rate of absorption after oral administration [40,44,45], which may explain the observed results.

Although oxytetracycline was one of the first antibiotics from the tetracycline group to be produced, there is a lack of information in the scientific literature on its pharmacokinetics in avian species [46,47].

Because of the characteristic chemical structure of tetracyclines, their absorption via the intestinal tract and their pharmacokinetic properties may be altered dramatically when these drugs are administered with food or substances that increase the stomach pH or contain divalent or trivalent cations, such as calcium, magnesium, manganese, aluminum, zinc, iron and bismuth, that support the formation of chelating complexes [48]. Due to its capacity to bind to divalent and trivalent cations, the highest concentration of oxytetracycline is found in the bones of broiler chickens submitted to pharmacological treatment with this antibiotic. Thus, the bone may also be considered a target tissue for monitoring oxytetracycline in poultry in addition to muscle, which is recommended by Regulation (EU) No. 37/2010 as a target tissue [23,49,50]. Odore et al. (2015) found concentrations of oxytetracycline below the MRL (100 μg kg^-1^) in muscle samples obtained from broiler chickens treated with this antibiotic [50].

In the current study, residues of enrofloxacin, ciprofloxacin and sulfaquinoxaline were present at concentrations higher than the MRL of each drug up to day 36 of breeding, i.e., up to two days after discontinuation of the treatment, and at the 38^th^ day of breeding, the observed concentrations were below the LOQ of the method. Residues of oxytetracycline at concentrations higher than the MRL were only observed during the period of treatment of the poultry. Technological advances in genetics and nutrition have allowed broiler chickens to attain high feeding efficiency and growth rates, such that they can reach the optimal slaughter weight within a few weeks [51]. The rapid muscle development and accelerated rate of metabolism of broiler chickens may contribute to the rapid elimination of the drug residues from the chicken muscles [52]. Thus, the findings of this study may reflect the high rate of metabolism of broilers and its effects on the pharmacokinetic properties of drugs, such as absorption, bioavailability, distribution, biotransformation and excretion.

An analytical method using UPLC-MS/MS was validated in this study, and its use was found to be adequate for the detection and quantification of antimicrobial residues in broiler chicken muscle. The depletion study demonstrated that residues of the antimicrobials enrofloxacin, ciprofloxacin, sulfaquinoxaline and oxytetracycline are rapidly metabolized from the broiler muscles, as the residue levels were lower than the LOQ of the method after four days of withdrawal for all of the studied analytes.
